# A *Drosera*-bioinspired hydrogel for catching and killing cancer cells

**DOI:** 10.1038/srep14297

**Published:** 2015-09-23

**Authors:** Shihui Li, Niancao Chen, Erin R. Gaddes, Xiaolong Zhang, Cheng Dong, Yong Wang

**Affiliations:** 1 Department of Biomedical Engineering, College of Engineering, Pennsylvania State University, University Park, Pennsylvania 16802-6804

## Abstract

A variety of bioinspired materials have been successfully synthesized to mimic the sophisticated structures or functions of biological systems. However, it is still challenging to develop materials with multiple functions that can be performed synergistically or sequentially. The purpose of this work was to demonstrate a novel bioinspired hydrogel that can interact with cancer cells, functionally similar to *Drosera* in catching and killing prey. This hydrogel had two layers with the top one functionalized with oligonucleotide aptamers and the bottom one functionalized with double-stranded DNA. The results show that the top hydrogel layer was able to catch target cells with high efficiency and specificity, and that the bottom hydrogel layer could sequester doxorubicin (Dox) for sustained drug release. Importantly, the released Dox could kill 90% of the cells after 1-h residence of the cells on the hydrogel. After the cell release, this bifunctional hydrogel could be regenerated for continuous cell catching and killing. Therefore, the data presented in this study has successfully demonstrated the potential of developing a material system with the functions of attracting, catching and killing diseased cells (e.g., circulating tumor cells) or even invading microorganisms (e.g., bacteria).

Development of materials for mimicking the sophisticated structures or functions of biological systems not only creates opportunities to gain deeper understanding of biological mechanisms^1^,^2^,^3^,^4^,^5^,^6^, but also holds the potential for discovering new applications^7^,^8^,^9^,^10^,^11^,^12^,^13^,^14^,^15^,^16^,^17^,^18^,^19^. For instance, the water-repellent surface structure of the lotus leaf has inspired the development of lotus-leaf-like nanostructured materials^7^,^8^; and the autonomic healing function of bone after fracture has inspired the formulation of composites that are able to release healing agents upon crack instrusion^17^,^18^,^19^. These bioinspired materials have been under rigorous investigation for various promising applications such as synthesis of super-hydrophobic materials and self-healing concrete. However, successful efforts have been primarily focused on the development of materials with the ability to mimic the singular functions of biological systems. It is still challenging to develop bioinspired materials with multiple functions that can be performed synergistically or sequentially.

The purpose of this work was to explore a hydrogel with the potential of mimicking the functions of *Drosera* in catching and killing prey (Fig. 1A). *Drosera* is a genus of carnivorous plants, whose leaves have tentacles covered with adhesive secretions^20^,^21^,^22^,^23^. When flying prey touch the leaves, they will be captured by the sticky tentacles^24^,^25^,^26^. In addition to presenting an adhesive surface to the environment, this plant releases digestive enzymes such as esterase, peroxidase, and protease on its leaves^27^. These enzymes can further digest and kill the captured prey. Since an unhealthy human body may have circulating diseased cells (e.g., cancer cells) or invading microorganisms (e.g., bacteria)^28^,^29^,^30^,^31^,^32^, the development of a bioinspired material with the functions of catching targets and releasing toxic drugs to destroy the targets may lead to potential biomedical applications. Thus, we were inspired to develop a novel hydrogel with the target-catching and drug-releasing functions.

This bifunctional hydrogel was made of two hydrogel layers (Fig. 1B). Both layers were functionalized with oligonucleotides. The top layer hydrogel was functionalized with nucleic acid aptamers. Nucleic acid aptamers are single-stranded oligonucleotides that can be, in principle, selected from oligonucleotide libraries with high affinities and specificities against any target molecules or cells^33^,^34^,^35^. Since aptamers are tolerant of harsh chemical conditions, they can be immobilized to a substrate without losing their binding capabilities^36^,^37^,^38^,^39^. Thus, aptamers were used to functionalize the top layer hydrogel for catching target cells via aptamer-mediated cell recognition. The chemical incorporation of aptamers into the hydrogel was evaluated with fluorescence imaging. The aptamer-mediated cell catch was studied by measurement of the kinetics of cell binding onto the top layer hydrogel. The bottom layer hydrogel was chemically functionalized with a double-stranded DNA that was used as an affinity site for sequestration of small toxic drugs. Thus, the drugs in the hydrogel could be locally released in a sustained manner. Drug sequestration and release were characterized with fluorescence spectroscopy. The function of the released drugs in killing the cells was examined with the cell viability assays.

## Results and Discussion

### Synthesis and characterization of the bilayer hydrogel

**Figure 2:**
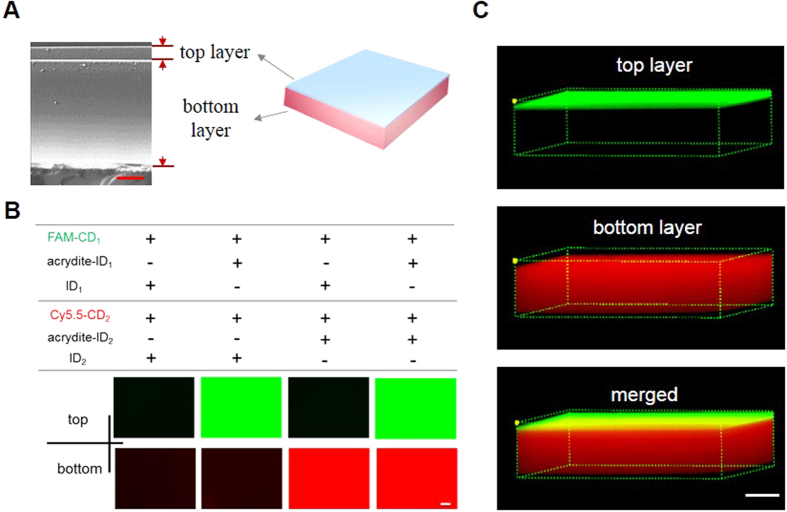
Characterization of the hydrogel. (**A**) SEM image of the hydrogel. Scale bar: 50 μm. (**B**) Fluorescence images of the hydrogel after treated with FAM-CD_1_ and Cy5.5-CD_2_. The green fluorescence was emitted by FAM-CD_1_ from the top layer hydrogel and the red fluorescence was emitted by Cy5.5-CD_2_ from the bottom layer hydrogel. All of the hydrogel samples were washed thoroughly before fluorescence imaging. Scale bar: 100 μm. (**C**) Confocal images of the hydrogel treated with FAM-CD_1_ and Cy5.5-CD_2_. The three demonsional image was generated by FV10-ASW version 3.0 software. Scale bar: 100 μm.

The bifunctional hydrogel was synthesized with two layers via two-step free radical polymerization (Figure S1). The top layer was ~25 μm thick and the bottom one was ~200 μm thick (Fig. 2A). During free radical polymerization, the immobilizing DNAs (IDs) including ID_1_ and ID_2_ were chemically incorporated into the top and bottom layers, respectively. To demonstrate the success of chemical incorporation of IDs into the hydrogel and intermolecular hybridization between the IDs and their complementary DNAs (CDs), the hydrogels were treated with FAM-CD_1_ and Cy5.5-CD_2_, washed thoroughly and examined under fluorescence microscopy. The hydrogels prepared with ID_1_ and/or ID_2_ without acrydite exhibited negligible fluorescence signals of Cy5.5 and/or FAM (Fig. 2B). In contrast, the hydrogels prepared with acrydite-modified ID_1_ and/or ID_2_ exhibited strong fluorophore signals. These results demonstrate that acrydite-modified ID_1_ and ID_2_ could be chemically incorporated into the two layers via free radical polymerization, and that IDs and CDs formed stable complexes via sequence-specific intermolecular hybridization. An in-depth observation of the bilayer hydrogel with confocal microscopy confirmed the incorporation and hybridization of the oligonucleotides (Fig. 2C). The two hydrogel layers played two different roles: the top one was used to present the hybridized aptamer (i.e., the ID_1_-CD_1_ complex) for cell catch and the bottom one was used to accommodate the drug intercalating double-stranded DNA (i.e., the ID_2_-CD_2_ complex) for Dox loading and release.

### Examination of cell catch on the hydrogel

**Figure 3:**
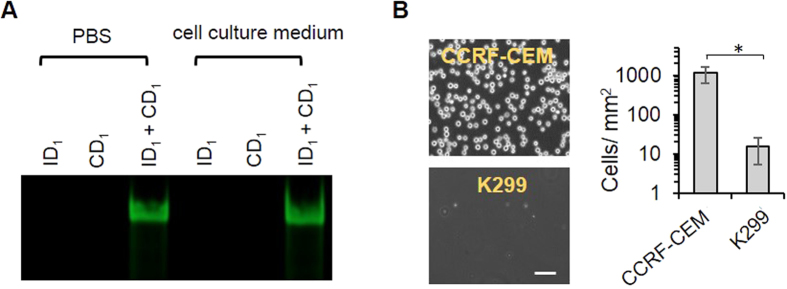
Examination of specific cell catch. (**A**) Gel image showing ID_1_-CD_1_ hybridization in two different solutions. (**B**) Imaging and quantitative analysis of cell catch on the hydrogels. The CCRF-CEM (target) cells and K299 (control) cells were incubated on the hydrogel surface for 1 h. Quantitative analysis was performed by the examination of three areas randomly chosen from each image. Cell numbers were analyzed by ImageJ. Scale bar: 50 μm. *P < 0.05; n = 3.

To catch target cells in a biologically relevant environment, the hybridized aptamer needs to have stability. Thus, the hybridized aptamer was examined using gel electrophoresis after it was incubated in the cell culture medium for 1 h. The gel electrophoresis image shows that the fluorescence intensity of the hybridized aptamer incubated in PBS and the cell culture medium was virtually the same (Fig. 3A), demonstrating that the aptamer complex exhibited stability during the 1-h incubation in the cell culture medium. The model aptamer used in this work was originally selected via a cell-SELEX procedure and it can bind the CCRF-CEM cell line with high specificity^40^. We incubated the CCRF-CEM cells with the hydrogel displaying he hybridized aptamer to test whether the hydrogel could catch the cells. Approximately 1,200 CCRF-CEM cells per mm^2^ were observed on the CD_1_ functionalized hydrogel whereas only ~10 control cells per mm^2^ was observed on the hydrogel (Fig. 3B). Thus, the data show that the top layer hydrogel with the hybridized aptamer has the ability to induce cell-type-specific catch in the biologically relevant environment.

While other affinity ligands such as antibodies and peptides may be applied to functionalize the top layer hydrogel to acquire the cell-catching function^41^,^42^, we chose to use nucleic acid aptamers to develop the top hydrogel layer for several reasons. Nucleic acid aptamers are selected in an *in vitro* environment^43^,^44^. They have high binding affinities and specificities against their targets owing to the rigorous procedure of aptamer selection. They are small in size and can be facilely synthesized using standard chemical methods. They are also tolerant of harsh chemical and physical conditions and suitable for chemical incorporation into a material^45^. These advantages distinguish nucleic acid aptamers from other ligands in the functionalization of the hydrogel.

A number of elegant material systems have been successfully developed with great potential to capture circulating tumor cells from blood for cell separation or cancer diagnosis^46^,^47^,^48^. However, most of them are made of hard materials presenting a rigid interface between the materials and the cells. By contrast, hydrogel is a soft material with many properties similar to human tissues. In addition, it is more flexible to conjugate affinity ligands to hydrogels or optimize hydrogels for controlled drug release in comparison to hard materials. Thus, hydrogels were used in this work to catch cancer cells and control drug release (next section).

### Examination of drug loading and release

**Figure 4:**
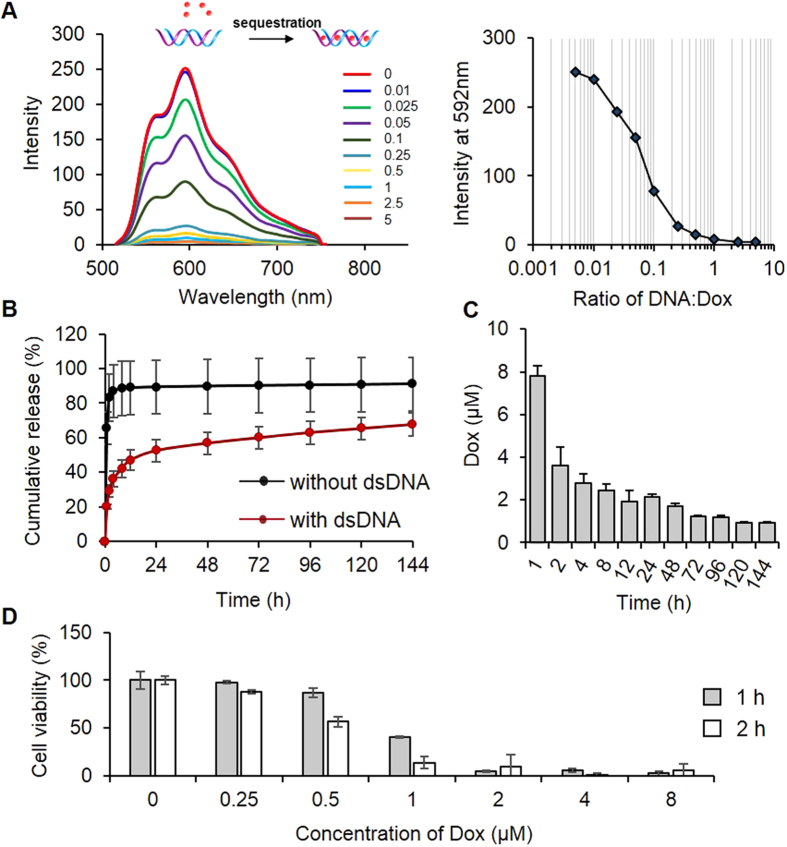
Sustained Dox release from the hydrogel. (**A**) Characterization of Dox (5 μM) sequestration by dsDNA using fluorescence spectroscopy. The labeling numbers from the top to the bottom indicate the molar ratios of DNA: Dox. The quantitative relationship between Dox and dsDNA is shown by the peak emission intensity at 592 nm versus the molar ratio. (**B**) Kinetics of cumulative Dox released from the hydrogel with or without the chemically conjugated dsDNA. (**C**) Concentrations of Dox released from the hydrogel with the chemically conjugated dsDNA at each sampling time points. (**D**) Effect of the Dox concentration on the viability of CCRF-CEM cells that were treated by Dox for either 1 h or 2 h.

To study the function of the bottom layer hydrogel in drug loading and release, Dox was used as a model drug. With the increase of the concentration of the ID_2_-CD_2_ complex, the fluorescence intensity of the Dox solution was decreased (Fig. 4A). The fluorescence intensity was decreased by 50% and 94% when the molar ratio was increased to ~0.07 and 0.5, respectively. Since the intercalation of Dox into double-stranded DNA leads to the quenching of Dox fluorescence^49^, the result indicates that Dox would be effectively loaded into the DNA-functionalized hydrogel. To directly illustrate this point, we incubated the hydrogel in the Dox solution for 24 h and subsequently transferred it into DPBS for the release test. The native hydrogel (without DNA) released 65.5% Dox during the first 1 h (Fig. 4B). The cumulative Dox release reached 86.8% and the release reached the plateau approximately after 4 h. In contrast, 19.8% and 36.0% of Dox was released from the dsDNA-functionalized hydrogel at 1 and 4 h, respectively. These data show that the incorporation of the ID_2_-CD_2_ complex was able to significantly reduce the initial burst release. Moreover, the release of Dox from the ID_2_-CD_2_ complex-functionalized hydrogel exhibited a pseudo-linear release profile after 4 h. Specifically, the concentrations of Dox in the release media were higher than or close to 1 μM during the entire 144 h test and were above 2 μM within the first 48 h (Fig. 4C). Since the function of the bottom layer hydrogel depends on whether the released Dox could kill the cells on the top layer hydrogel, we examined the relationship of cell viability versus the concentration of Dox. The results show that the viability of the cells was decreased to 36.7 and 4.9% after the 1-h exposure to 1 and 2 μM of Dox, respectively (Fig. 4D). Increasing the exposure time of cells to Dox could significantly decrease the cell viability in the low Dox concentration groups, e.g., 1 μM.

In this work, we applied the ID_2_-CD_2_ complex to functionalize the bottom layer hydrogel to achieve the sustained Dox release. Other methods may be used to optimize and improve the bottom layer hydrogel for a longer period of drug release. For instance, biodegradable microparticles with the ability to control the release of drugs for weeks or months have been used to functionalize hydrogels^50^,^51^,^52^. Such a method may be applied to develop the bottom hydrogel. The bottom hydrogel may also be functionalized with enzymes that can covert prodrugs into drugs for a long-term sustained release^53^. In addition to using hydrogels to control drug release, a variety of other polymeric systems have been studied for long-term sustained drug release^54^,^55^. Those sustained release systems can, in principle, be used to replace the bottom hydrogel for the optimization of the sustained drug release.

### Examination of cell catch and killing on the bifunctional hydrogel

**Figure 5:**
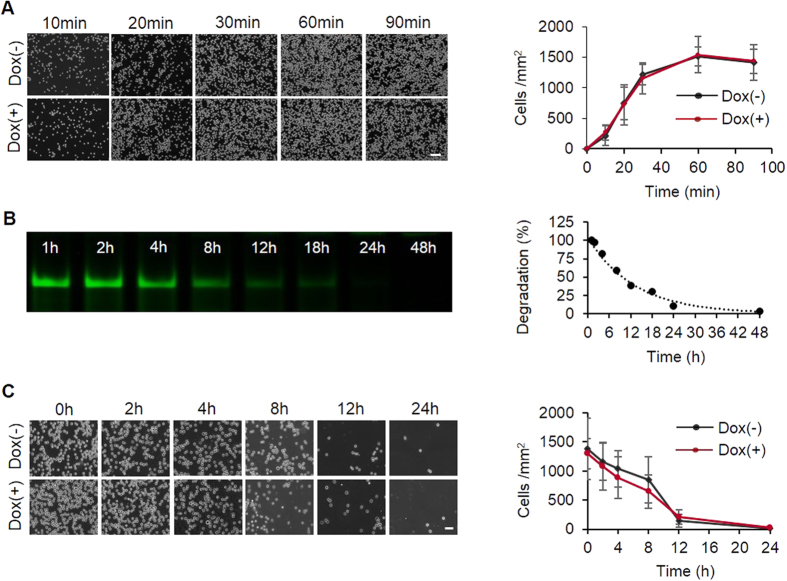
Cell catch and release kinetics on the hydrogel. (**A**) Kinetics of CCRF-CEM cell catch on hydrogels in the presence or absence of Dox. Scale bar: 100 μm. (**B**) Degradation of dsDNA in the culture medium. The electrophoretic gel image was analyzed with the software of Maestro imaging system to generate the profile of dsDNA degradation versus time. (**C**) Kinetics of CCRF-CEM cell release from the hydrogels in the presence or absence of Dox. For the cell release study, the starting 0 h indicates 1 h after cell catch. Scale bar: 50 μm.

After demonstrating the cell-catching and drug-releasing functions of each hydrogel layer, we examined cell behavior on the aptamer-functionalized drug-loaded hydrogel. The number of cells increased linearly during the first half hour and gradually reached a plateau between 0.5 and 1.5 h (Fig. 5A). Moreover, the results show that the presence or absence of Dox did not affect the outcome of cell catch, suggesting that the presence of drugs did not affect the cell-binding functionality of the hybridized aptamer. In addition to the examination of cell catch during the first 1.5 h, we examined the cell release kinetics. With the increase of the incubation time, the ID_1_-CD_1_ complex was gradually degraded (Fig. 5B). As a result, the attached cells were released from the hydrogel surface. The density of the cells on the hydrogel was decreased to 50.1 and 99.8% after 8 and 24 h incubation in the presence of serum (Fig. 5C). The results also show that the presence or absence of Dox did not affect the kinetics of cell release (Fig. 5C).

**Figure 6:**
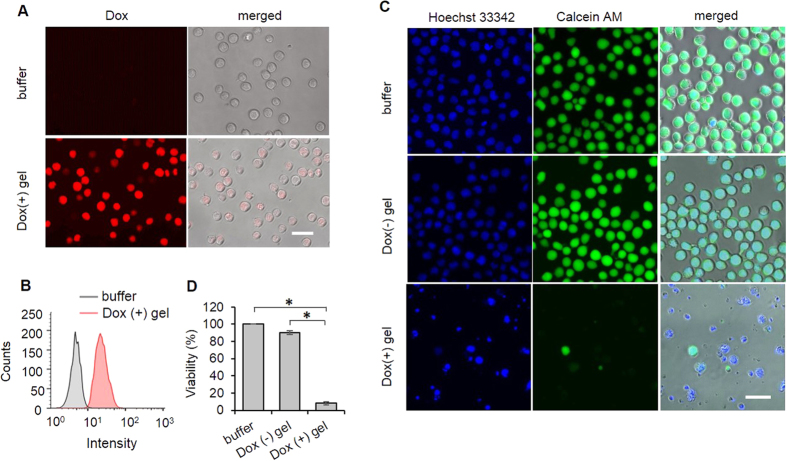
Examinations of drug uptake and cell viability. (**A**) Fluorescence images of CCRF-CEM cells after 1-h residence on the Dox-releasing hydrogel. The cells were harvested and analyzed after 1-h residence on the hydrogel. Scale bar: 20 μm. (**B**) Flow cytometry histogram. The CCRF-CEM cells were harvested and analyzed after 1-h residence on the hydrogel. (**C**) Examination of cell viability via the cell staining assay. The harvested CCRF-CEM cells were cultured in the cell culture medium for 48 h and then stained with Hoechst 33342 and Calcein AM. Hoechst 33342 was used to stain the nuclei of cells; Calcein AM was used to stain live cells. Scale bar: 20 μm. (**D**) Analysis of cell viability with the MTS assay. *P < 0.05; n = 3.

Since the kinetic profiles of cell-hydrogel interactions show that the cell density reached a plateau at 1 h, we harvested the cells from the hydrogel for examining cellular Dox uptake using both fluorescence microscopy and flow cytometry. The fluorescence images show that the cells on the hydrogel exhibited a strong fluorescence signal from Dox (Fig. 6A). The imaging result was confirmed by the flow cytometry (Fig. 6B). Thus, the data show that the cells were able to take up a significant amount of Dox released from the bottom layer hydrogel within the first 1 h before the onset of cell release.

After the examination of Dox uptake by the cells, the effectiveness of Dox-mediated cell killing was investigated via the cell viability assays. As shown in the fluorescence images, the cells without Dox treatment could maintain the same bioactivity and morphology as those intact cells (i.e., cells incubated in normal cell culture medium) (Fig. 6C). In contrast, most of the cells harvested from the Dox-releasing hydrogel died with irregular shapes. The quantitative data showed that the viability of these cells was decreased to less than 10% (Fig. 6D), suggesting that the short duration of drug uptake was sufficient to kill the majority of the cells. To illustrate the effect of the residence time on drug uptake, we harvested the cells from the hydrogel at the different time points and characterized their Dox uptake via flow cytometry. The results show that the amount of Dox uptake increased with the time (Figure S2A). The 12-h exposure of the cells to Dox resulted in cellular fragmentation into membrane-bound apoptotic bodies, which is a hallmark of cell apoptosis and death (Figure S2B).

### Evaluation of the sustainability of catching and killing cells

**Figure 7:**
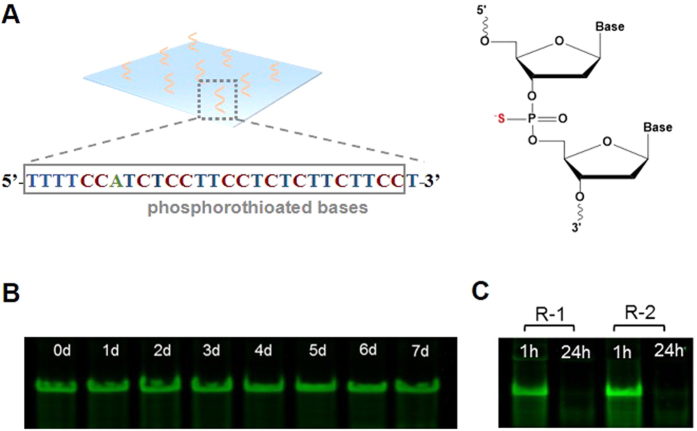
Examination of the stability of phosphorothiolated DNA. (**A**) Chemical structure of ID_1_ on the top layer hydrogel. The backbone of ID_1_ has the phosphorothioate bond resistant to nuclease degradation. The 3′ ends was modified with the inverted T. (**B**) Gel electrophoresis image demonstrating the resistance of ID_1_ against nuclease degradation during the 7-day degradation test. After ID_1_ was degraded in the cell culture medium for different days, CD_1_ was added into ID_1_ solutions before gel electrophoresis. (**C**) Gel image of the ID_1_-CD_1_ duplex before and after the incubation in the cell culture medium in a two-round test. The duplex was incubated in the cell culture medium for 24 h. This procedure was repeated after the duplex solution was added with fresh CD_1_.

**Figure 8:**
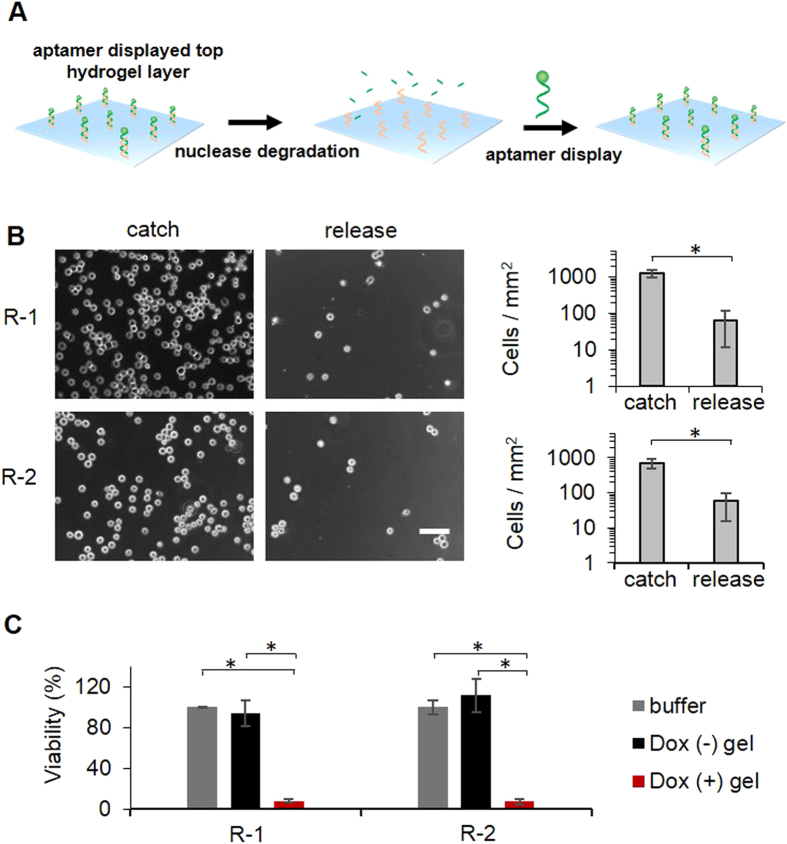
Evaluation of sustained cell catch and killing. (**A**) Schematic illustration of alternate aptamer display and nuclease degradation on the top layer hydrogel. ID_1_ is nondegradable owing to the full modification of its backbone whereas unmodified CD_1_ is degradable. (**B**) Microscopic images and quantitative analysis of CCRF-CEM cell catch and release in a two-round test. Scale bar: 50 μm. (**C**) Analysis of cell viability. *P < 0.05; n = 3.

After catching prey, the tentacles of *Drosera* can return to their original position to continuously catch and kill new prey. Since aptamer degradation occurred during the procedure of cell release (Fig. 5), the hydrogel may lose the ability to catch target cells without fresh aptamers displayed on the top hydrogel layer. To regenerate the surface with fresh aptamers for a new batch of cell catch, we used a chemically modified ID_1_ for the functionalization of the top hydrogel layer. One of the non-bridging oxygen in each phosphate was replaced by sulfur (Fig. 7A). The image shows that ID_1_ degradation was undetectable during a 7-day degradation test (Fig. 7B), indicating that the sulfurization of the backbone stabilized ID_1_ in the serum. Thus, chemically modified ID_1_ on the top layer hydrogel was able to hybridize with fresh aptamers after the original ones were degraded (Fig. 7C). Resultantly, the cell-catching function of the hydrogel can be regenerated. Indeed, the regenerated hydrogel was able to sustainably catch and kill cells (Fig. 8). In addition to the replacement of the non-bridging oxygen with sulfur, there is a diverse array of other chemical methods available to stabilize oligonucleotides against nuclease degradation^56^,^57^. Those methods can also be used to optimize the chemical modification of ID_1_.

The major focus of the current work was to develop a bioinspired hydrogel with multiple functions. However, one may inquire whether this material can be used in the human body for catching and killing diseased cells or invading microorganisms. We envision that this multifunctional material holds potential of treating human diseases for the following reasons. First, in nature, circulating cancer cells can be captured onto the endothelium surface during circulation via molecular recognition^58^. Similarly, invading bacteria in the circulation can bind to implant surfaces^59^. These biological effects suggest that a material with the function of cell recognition would be able to catch target cells or microorganisms in an *in vivo* setting. Second, the human body is virtually a closed system for circulation. Thus, the multifunctional material has multiple opportunities to catch the circulating cells in a given period of time. This differs from *in vitro* diagnostic tools (e.g., microfluidic devices for separation of circulating cancer cells) that usually need to capture target cells during the first pass of the cells over the surface of the affinity material. Third, while high shear stress is not favorable for cell catch on a surface, there are many locations (e.g., veins or lymphatic vessels) in the human body where the shear stress of the circulation is low. Fourth, the multifunctional material can be designed in diverse forms such as a depot or a conduit to satisfy the different requirements of implantation in appropriate locations. Certainly, more work needs to be performed in the future to demonstrate the feasibility of using this multifunctional bioinspired material *in vivo*.

## Conclusion

In summary, we synthesized a novel bioinspired hydrogel functionally similar to *Drosera* in catching and killing prey. This hydrogel is able to catch cancer cells effectively and release drugs locally to kill the cells. In addition, it can repetitively display the cell-catching aptamer on the surface. As a result, the sustained drug release from this bioinspired hydrogel can continuously kill cancer cells. The functions of this material will be further studied in an *in vivo* setting. Future work may also be performed to integrate the controlled chemokine release function into the hydrogel for attracting cancer cells. Thus, the *Drosera*-bioinspired hydrogel will have the ability to attract, catch, and kill target diseased cells or even invading microorganisms for biomedical applications.

## Methods

### Chemical reagents

Doxorubicin, 3-(trimethoxysilyl) propyl methacrylate (TMSPM), and magnesium chloride (MgCl_2_) solution (1.0 M) were obtained from Sigma Aldrich (Louis, MO). Dulbecco’s phosphate buffered saline (DPBS), SYBR-Safe, Calcein AM, and Hochest 33342 were purchased from Invitrogen (Carlsbad, CA). RPMI-1640 was purchased from ATCC (Manassas, VA). Fetal bovine serum (FBS) and the penicillin/streptomycin solution were purchased from Hyclone (Logan, UT). The CellTiter 96 AQueous One Solution Cell Proliferation Assay (MTS) kit was from Promega (Madison, WI). Nucleic acid oligonucleotides (Table 1 in supporting information) were synthesized by Integrated DNA Technologies (Coralville, IA). Other chemicals were purchased from Fisher Scientific (Suwanee, GA).

### Synthesis of the bilayer hydrogels

The bilayer hydrogels were synthesized on the surface of silanized glass slides with a dimension of 7.5 × 7.5 mm^2^. The silanization of glass slides was performed according to the method described previously^35^. In brief, the NaOH-treated glass slides were reacted with the TMSPM solution for 5 min and subsequently washed with ethanol and dried in the air. To synthesize the bottom hydrogel, a pregel solution was prepared with ammonium persulfate (APS, 0.5 μL, 10% w/v), N,N,N’,N’-tetramethylenediamine (TEMED, 0.5 μL, 10% v/v), acrylamide solution (19 μL, 10% w/v) and acrydite-modified ID_1_ (125 μM). This pregel solution was transferred onto the surface of silanized glass and covered with coverslip. After the hydrogel was cured for 2 h, the coverslip was gently removed from the cured hydrogel. The hydrogel on the silanized glass square was washed in PBS for 1 h to remove the unreacted molecules. The top layer hydrogel was synthesized on the bottom hydrogel using a similar procedure. The pregel solution containing acrylamide solution (4 μL, 10% w/v), acrydite-modified ID_2_ (100 μM), APS (0.25 μL, 10% w/v), and TEMED (0.25 μL, 10% v/v) was sandwiched between the bottom hydrogel and a piece of supporting naked glass slide. After cured for 2 h, the bilayer hydrogel was flipped off the supporting glass slide and washed thoroughly with PBS.

### Gel electrophoresis

Gel electrophoresis was used to examine intermolecular hybridization and degradation kinetics. To test intermolecular hybridization, ID_1_, ID_2_ were incubated with complementary sequences at a molar ratio of 1:1 in PBS for 1 h at room temperature. To study the degradation of oligonucleotides, the oligonucleotides either in a single-stranded or hybridized form were incubated in the culture medium (RPMI 1640 medium, 10% FBS, and 100 IU/mL penicillin-streptomycin) at 37 °C. At predetermined time points, 25 μL aliquot of the mixture was collected and stored at −20 °C. The solution of single-stranded oligonucleotides was mixed with the complementary sequences prior to the analysis. To examine the functionality of ID_1_ in repeatedly hybridizing CD_1_, a solution containing ID_1_ (0.8 μM) and CD_1_ (0.8 μM) was incubated in the cell culture medium at 37 °C. At 1 h and 24 h, 25 μL of the solution was collected and stored at −20 °C. The solution collected at 24 h was also mixed with the fresh CD_1_ solution (0.8 μM). The samples were collected at 1 h and 24 h. The collected samples were loaded into a 10% polyacrylamide gel. The electrophoresis was run in the TBE buffer (89 mM tris-borate, 89 mM boric acid, and 2 mM EDTA; pH 8.2) at 140 V for 40 min using a Mini-PROTEAN Tetra Cell system (Bio-Rad, Hercules, CA). The gel was stained in SYBR-Safe solution for 30 min and imaged with a green fluorescence filter using a CRI Maestro EX System (Woburn, MA). The images were analyzed using the software provided by the supplier.

### SEM imaging of the hydrogels

The bilayer hydrogel was treated with a series of ethanol solutions (50%, 60%, 70%, 80%, 90%, and 100%) for gradient dehydration. After the dehydration, the hydrogels were dried in air and coated with gold. The cross-section of the hydrogels were imaged under a Hitachi S-3500N scanning electron microscope (Hitachi, Ltd, Tokyo, Japan). SEM imaging was acquired at an accelerating voltage of 20 kV.

### Fluorescence imaging of the hydrogels

The hydrogels prepared with ID_1_ and ID_2_ (with or without acrydite) were incubated with 20 μL Cy5.5-CD_2_ solution (125 μM) for 2 h. After incubated in PBS for 1 h washing, the hydrogels were incubated with 20 μL FAM-CD_1_ (20 μM) for 1 h. The hydrogels were washed thoroughly in PBS to remove the free fluorophore-labeled DNA molecules. The hydrogels were imaged under the Olympus FV1000 laser scanning confocal microscope (Olympus America Inc., Melville, NY) with a 20X objective. Blue Argon (488 nm) and red HeNe (633 nm) lasers were used to catch the signals of Cy5.5 and FAM, respectively. The hydrogel was scanned from the top where fluorescence appeared to the bottom until fluorescence disappeared. The images was analyzed using FV10-ASW version 3.0 software. In addition to the in-depth observation of the fluorescence signals, the hydrogels were also imaged under a fluorescence microscope (Olympus IX73, Melville, NY) with a 10X objective to examine the incorporation of ID_1_ and ID_2_. The fluorescence signals were recorded by using two sets of fluorescence filter cubes at 480 ± 20/535 ± 25 nm (Ex./Em.) and 620 ± 30/700 ± 40 nm (Ex./Em.).

### Dox loading and release

The hydrogels were incubated with the CD_2_ solution of 20 μL (125 μM in DPBS) at room temperature for 2 h and washed with PBS thoroughly. The hydrogels were incubated with 40 μL of DPBS containing 5 mM MgCl_2_ and Dox (500 μM) at 4 °C for 24 h. To examine Dox release, the Dox-loaded hydrogels were incubated in 500 μL of DPBS with a shaking speed of 70 rpm at 37 °C. At predetermined time points, the supernatants were collected and replaced with 500 μL of fresh DPBS. The collected supernatants were transferred into a fluorescence 96-well plate (Perkin Elmer, Waltham, MA) and measured by a Tecan F200 Pro Micro-plate reader (Tecan US Inc., San Jose, CA) at 592 nm.

### Cell culture

CCRF-CEM cell (CCL-119, human T lymphocytic leukemia cell line) was obtained from ATCC (Manassas, VA); K299 (Karpas 299, human T cell lymphoma cell line) was purchased from Sigma-Aldrich. The cells were cultured in the cell culture medium in an incubator at 37 °C in a 5% CO_2_ atmosphere.

### Examination of the interactions between cells and the hydrogels

The hydrogel was washed in cell culture medium for 2 h and then incubated with 20 μL of CD_1_ solution (20 μM in cell culture medium) at 37 °C for 1 h for the immobilization of CD_1_ on the top layer hydrogel. The hydrogel was washed with 500 μL of culture medium for 10 min to remove free CD_1_ and then incubated with 500 μL of cell suspension (3 × 10^5^ cells in the cell culture medium supplemented with 5 mM MgCl_2_) in a 48-well plate at 37 °C for 1 h. The unbound cells were removed after the 48-well plate was gently shaken at 70 rpm for 1 min. Five hundred microliters of fresh cell culture medium was added into the plate and the hydrogel was incubated at 37 °C in the CO_2_ incubator for 24 h. At predetermined time points, the hydrogel was examined under the microscope. Three areas of the hydrogel surface were randomly selected for imaging. The cells in each area were enumerated using ImageJ. To examine the sustainability of using the hydrogel to catch cells, the whole procedure of immobilizing CD_1_ and capturing cells was repeated after the first round of cell catch and release.

### Examination of Dox uptake by cells

At predetermined time points after cell attachment onto the hydrogel, the cells were collected from hydrogel surface. After washing with 1 mL of DPBS twice, the collected cells were imaged using the fluorescence microscope with a red fluorescence filter (535 ± 25/610 ± 40 nm, Ex./Em.) and a 40X objective. The collected cells were also examined with a Coulter FC500 flow cytometer (Beckman Coulter, Miami, FL). A total of 5,000 events were counted for each group. The data were analyzed with FlowJo 7.0.

### SEM imaging of the cells on the hydrogel

The cells on the hydrogel surface were fixed in glutaraldehyde solution (3% in PBS) for 3 h. After rinsing with PBS, the samples were dehydrated in a series of ethanol solutions (50%, 60%, 70%, 80%, 90%, and 100%). Subsequently, the samples were dehydrated in Hexamethyldisilazane (HMDS) solutions (33%, 66%, and 100%) and dried in air overnight. The cells were coated with a gold layer. The SEM imaging was operated under a Hitachi S-3500N scanning electron microscope (Hitachi, Ltd, Tokyo, Japan) at an accelerating voltage of 15.0 kV.

### Analysis of cell viability

The cells were incubated in 8-well chamber slide with an initial concentration of 2.5 × 10^5^ cells/mL in the culture medium for 48 h and were then stained in the mixture of Hochest 33342 (2.5 μg/mL) and Calcein AM (1 μM) at 37 °C for 30 min. After the replacement of the staining medium with DPBS, the cells were imaged under the fluorescent microscope with two sets of fluorescence filter cubes (350 ± 25/460 ± 25 nm (Ex./Em.) and 480 ± 20/535 ± 25 nm (Ex./Em.) and a 40X objective. Quantitative analysis of the cell viability was performed using a CellTiter MTS cell assay kit. In brief, CCRF-CEM cells were collected from the hydrogel surface, seeded into 96-well plate with a population of 2.5 × 10^4^ cells in 100 μL of cell culture medium and cultured in CO_2_ incubator for 48 h. The CellTiter reagent (20 μL) was added to each well and incubated with the cells at 37 °C for 3 h. The absorbance at 490 nm was recorded using the Tecan Micro-plate reader. The absorbance in each group was normalized to the average absorbance of the group with intact cells to present the viability.

### Statistical analysis

Student’s t-test (two-sided) was used to determine the significance of results. P-value < 0.05 was considered as significant. Error bars in all figures represent standard deviations.

## Additional Information

**How to cite this article**: Li, S. *et al.* A *Drosera*-bioinspired hydrogel for catching and killing cancer cells. *Sci. Rep.* **5**, 14297; doi: 10.1038/srep14297 (2015).

## Supplementary Material

Supporting information

## Figures and Tables

**Figure 1:**
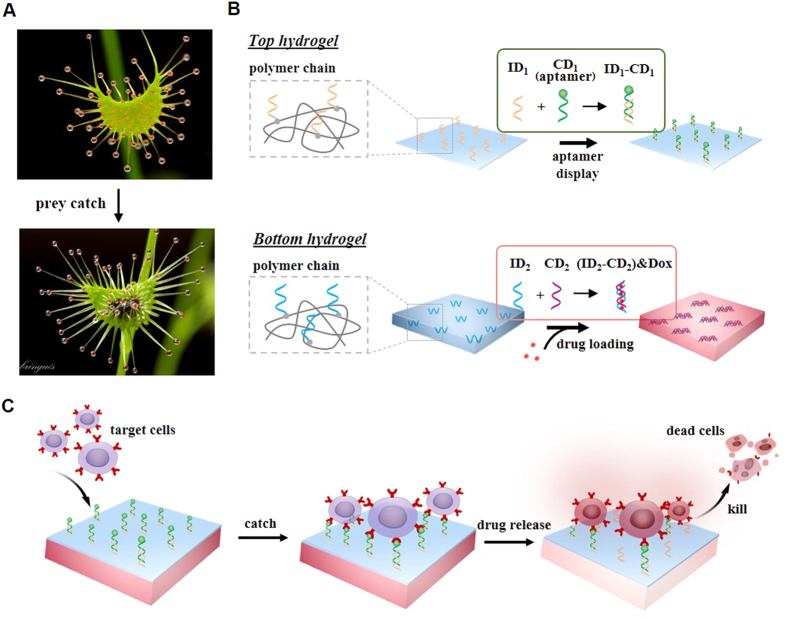
Conceptual illustration of *Drosera*-bioinspired hydrogel. (**A**) Photos of *Drosera* before and after the prey catch. (**B**) Schematic illustration of the bilayer hydrogel made of acrydite-DNA, acrylamide and bisacrylamide. ID_1_ and ID_2_ are chemically incorporated into the top and bottom hydrogel layers during free radical polymerization, respectively. ID_1_ hybridizes with CD_1_ on the top layer for catching target cells; and ID_2_ hybridizes with CD_2_ to sequester drug (Doxorubicin) in the bottom hydrogel. (**C**) Schematic illustration of cell catch and drug release for killing target cells on the hydrogel.
