# Supplementary material for: A Drosera-bioinspired hydrogel for catching and killing cancer cells
Source: Sci Rep. 2015 Sep 23;5:14297. doi: 10.1038/srep14297 (PMC4585793; doi:10.1038/srep14297)
Supplement: Supporting information [file srep14297-s1.pdf]

Supporting Information

# A *Drosera*-bioinspired hydrogel for catching and killing cancer cells

Shihui Li, Niancao Chen, Erin R. Gaddes, Xiaolong Zhang, Cheng Dong, Yong Wang*

Department of Biomedical Engineering, College of Engineering, Pennsylvania State University,

University Park, Pennsylvania 16802-6804

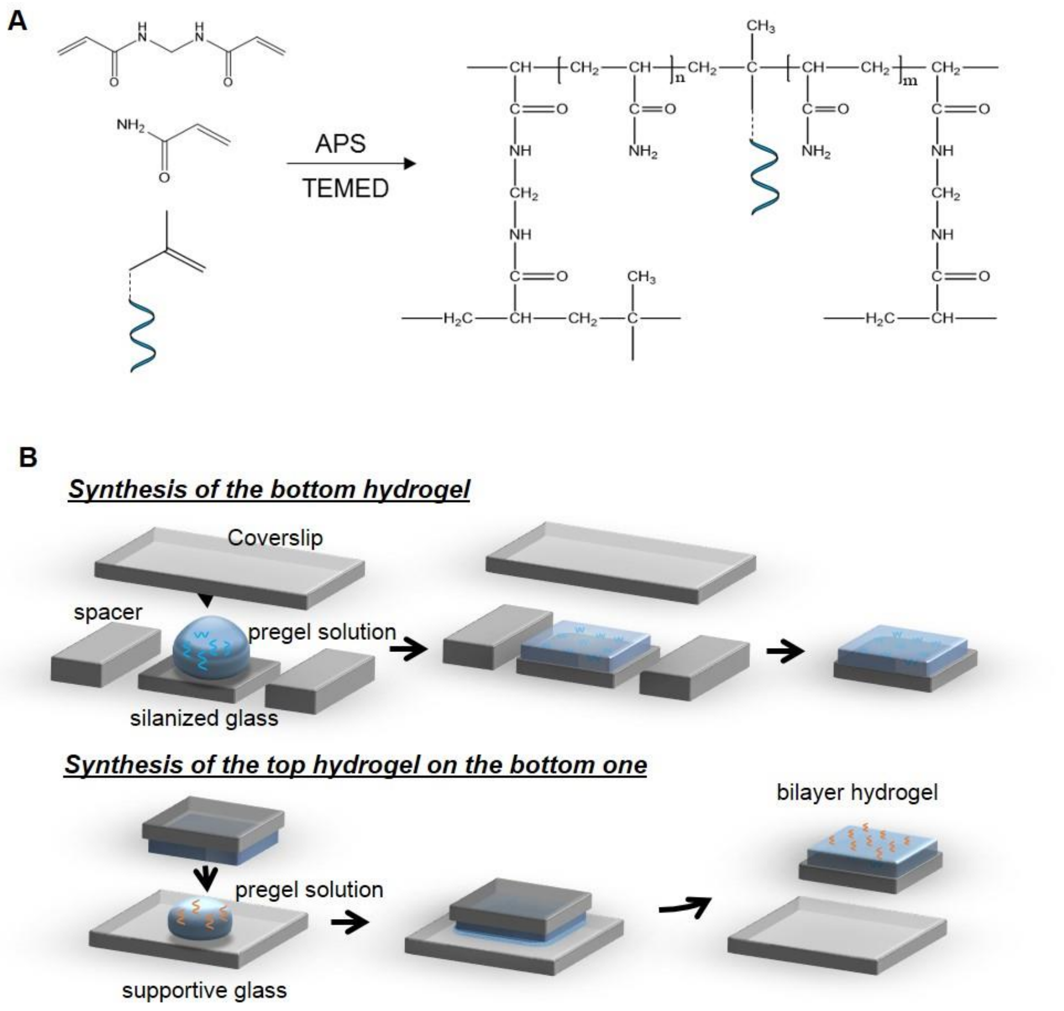


**Figure S1.** (**A**) Synthesis of the hydrogel. (**B**) Procedure of the bilayer hydrogel fabrication.

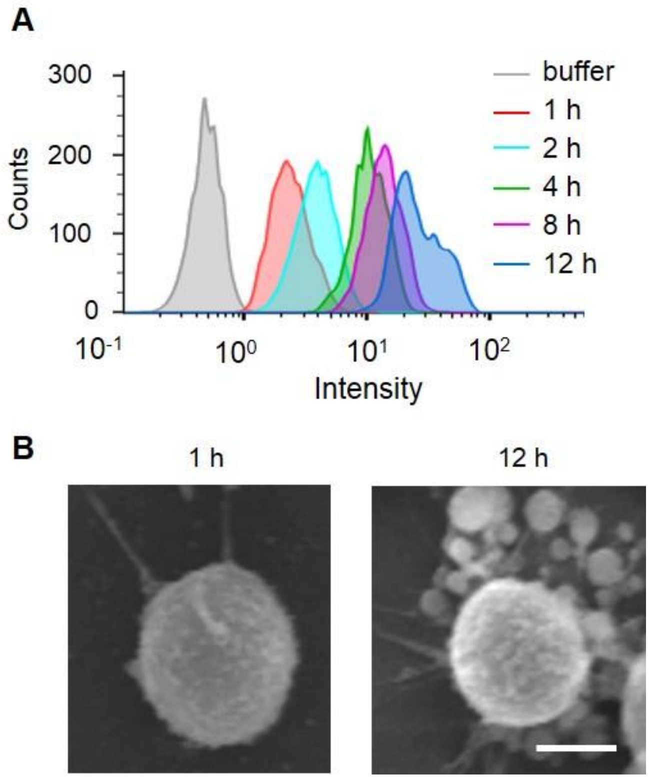


**Figure S2.** (**A**) Flow cytometry histogram showing the relationship of drug uptake versus time. Cells were harvested from the hydrogel at different cell residence time points. (**B**) SEM images of the cells on the Dox (+) hydrogel after 1-h and 12-h residence. Scale bars: 5 μm.

**Table 1.** DNA sequences.

| DNA | 5'-3' |
|---|---|
| *Acrydite*-$ID_1$ | *Acrydite*-T*T*T*T*C*C*A*T*C*T*C*C*T*T*C*C*T*C*T*C*T*T*C*T*T*C*C*T/3InvdT/ |
| $ID_1$ | T*T*T*T*C*C*A*T*C*T*C*C*T*T*C*C*T*C*T*C*T*T*C*T*T*C*C*T/3InvdT/ |
| $CD_1$ | TCTAACTGCTGCGCCGCCGGGAAAATACTGTACGGTTAGATATATAGG AAGAAGAGAGGAAGGAGATGGAA |
| FAM-$CD_1$ | *FAM*-TCTAACTGCTGCGCCGCCGGGAAAATACTGTACGGTTAGATATATAGGA AGAAGAGAGGAAGGAGATGGAA |
| *Acrydite*-$ID_2$ | *Acrydite*-GCAGCAGCAGCAGCAGCAAAA |
| $ID_2$ | GCAGCAGCAGCAGCAGCAAAA |
| $CD_2$ | TTTTGCTGCTGCTGCTGCTGC |
| Cy5.5-$CD_2$ | *Cy5.5*-TTTTGCTGCTGCTGCTGCTGC |

* *Phosphorothiolated base*

ID: *Immobilizing DNA*

CD: *complementary DNA*

$CD_1$ *is the aptamer for cell catch.*
